# Characterization of the ABC Transporter G Subfamily in Pomegranate and Function Analysis of *PgrABCG14*

**DOI:** 10.3390/ijms231911661

**Published:** 2022-10-01

**Authors:** Qing Yu, Jiyu Li, Gaihua Qin, Chunyan Liu, Zhen Cao, Botao Jia, Yiliu Xu, Guixiang Li, Yuan Yang, Ying Su, Huping Zhang

**Affiliations:** 1 College of Horticulture, Nanjing Agricultural University, Nanjing 210095, China; 2 Key Laboratory of Horticultural Crop Genetic Improvement and Eco-Physiology of Anhui Province, Key Laboratory of Fruit Quality and Developmental Biology, Institute of Horticultural Research, Anhui Academy of Agricultural Sciences, Hefei 230031, China

**Keywords:** pomegranate, seed hardness, inner seed coat, ABCG transporter, evolution, lignin biosynthesis

## Abstract

ATP-binding cassette subfamily G (ABCG) proteins play important roles in plant growth and development by transporting metabolites across cell membranes. To date, the genetic characteristics and potential functions of pomegranate ABCG proteins (PgrABCGs) have remained largely unknown. In this study, we found that 47 PgrABCGs were divided into five groups according to a phylogenetic analysis; groups I, II, III, and IV members are half-size proteins, and group V members are full-size proteins. *PgrABCG14*, *PgrABCG21*, and *PgrABCG47* were highly expressed in the inner seed coat but had very low expression levels in the outer seed coat, and the expression levels of these three *PgrABCG* genes in the inner seed coats of hard-seeded pomegranate ‘Dabenzi’ were higher than those of soft-seeded pomegranate ‘Tunisia’. In addition, the expression of these three *PgrABCG* genes was highly correlated with the expression of genes involved in lignin biosynthesis and hormone signaling pathways. The evolution of *PgrABCG14* presents a highly similar trend to the origin and evolution of lignin biosynthesis during land plant evolution. Ectopic expression of *PgrABCG14* in *Arabidopsis* promoted plant growth and lignin accumulation compared to wild type plants; meanwhile, the expression levels of lignin biosynthesis-related genes (*CAD5*, *C4H*, and *Prx71*) and cytokinin response marker genes (*ARR5* and *ARR15*) were significantly upregulated in transgenic plants, which suggests the potential role of *PgrABCG14* in promoting plant growth and lignin accumulation. Taken together, these findings not only provide insight into the characteristics and evolution of *PgrABCG*s, but also shed a light on the potential functions of *PgrABCG*s in seed hardness development.

## 1. Introduction

Pomegranate (*Punica granatum* L.) is a perennial fruit tree grown in regions with Mediterranean climates. It grows in countries such as Tunisia, Turkey, Spain, Egypt, Morocco, the United States, China, India, Argentina, Israel, and South Africa [1]. The nutritional and medicinal pomegranate fruits attract much interest from consumers [2,3]. The soft and juicy outer seed coat is the edible part of the pomegranate, while the lignified inner seed coat is hard, which affects the feeling of swallowing fresh fruits. The lignin accumulation in the inner seed coat of hard-seed pomegranate is significantly higher than that of soft-seed pomegranate [4]. Thus, genetic studies of inner seed coat development are appealing for facilitation of breeding and development of pomegranate cultivars with low seed hardness.

In pomegranates, the differentiation and expansion of seed coat cells occurs in the early stages of fruit development [5]. Secondary cell wall thickening and lignin accumulation presents in the inner seed coat at the later stage of fruit development, leading to the formation of seed hardness [4]. In plants, the staple biosynthetic pathway of lignin is well understood: at the first step, monolignols including *p*-coumaryl alcohol (H-type monolignol), coniferyl alcohol (G-type monolignol), and sinapyl alcohol (S-type monolignol), are produced by phenylpropanoid pathways within the plant cell; at the second step, the monolignols are transported across the plasma membrane to the cell wall; and at the last step, the monolignols are polymerized to form lignin under the action of peroxidase and laccase [6,7,8]. To date, many genes encoding key enzymes and transcription factors that are involved in the biosynthesis and polymerization of monolignols have been characterized [9,10,11,12,13]. Recently, studies have demonstrated that monolignols can be passively diffused from inside the cell out to the cell wall through lipid bilayers [14]. However, transporters responsible for active transport of monolignols remains elusive.

Phytohormones play important roles in regulating cell differentiation and lignin accumulation. Indole acetic acid (IAA) and gibberellic acid (GA) were confirmed to control the lignin formation in primary phloem fibers and in xylem of Coleus blumei stems [15]. In *Arabidopsis*, Abscisic acid (ABA) regulates secondary cell wall formation and lignin deposition [16]. The IAA and ABA responsive transcription factor *CgMYB58* upregulates lignin biosynthesis and triggers juice sac granulation in pummelo [17]. The differential distribution of IAA affects the lignin composition during the tilting response in Pinus radiata seedlings [18]. In carrots, exogenous cytokinin significantly upregulates most of the expression levels of lignin biosynthesis genes, causing elevated lignin accumulation in taproots [19]. Similarly, cytokinin elevated lignification and upregulate the expression level of several key genes related to lignin biosynthesis in bamboo [20,21]. These studies suggested that phytohormones may play important roles in regulating seed coat development, especially for lignification.

ABCG proteins are the G type of ATP-binding cassette (ABC) transporters, which can transport a variety of metabolites, including monolignols, hormones, and sterol glycosides. *ABCG* genes have been identified in many plants such as *Arabidopsis thaliana* [22], rice (*Oryza sativa*) [23], corn (*Zea mays*) [24], alfalfa (*Medicago truncatula*) [25], grapes (*Vitis vinifera*) [26], pineapple (*Ananas comosus*) [27], and tomato (*Solanum lycopersicum*) [28]. *AtABCG14* plays an important role in delivering cytokinin from the root to the shoot. Knocking out *AtABCG14* strongly impairs the plant’s growth and development, lignin synthesis and deposition are considerably delayed, and the numbers of lignified cells are substantially reduced in the interfascicular fibers and xylem bundles of the mutant plants [29,30]. In addition, AtABCG16, 25, 30, 31, 36, 37, and 40 are involved in hormone transport [31,32,33,34,35]. AtABCG9 functions in sterol glycoside transport in *Arabidopsis* [36]. In addition, *AtABCG9*, *AtABCG11*, and *AtABCG14* were reportedly required for vascular development in *Arabidopsis* [37]. AtABCG29 functions as a *p*-coumaryl alcohol exporter involved in lignin biosynthesis in *Arabidopsis* [38]. The expression levels of *AtABCG29*, *30*, *33*, *34*, and *37* were co-expressed with reference genes involved in lignification [39]. These studies imply the multifunctionality of ABCG transporters involving the translocation of diverse substrates.

In pomegranate genome, 47 *ABCG* genes were identified [40], but their genetic characteristics and potential functions remained unclear. In this study, integrated analyses of phylogenetic classification, gene and protein structures, expression patterns, and evolution were conducted to investigate the characteristics of the *PgrABCG* genes. We cloned one candidate gene, called *PgrABCG14*, to investigate its biological function by ectopic expression in *Arabidopsis*. This study not only provides insight into *PgrABCG* characteristics, but also provides valuable references for further studies on the potential function of *PgrABCG* genes involved in inner seed coat development, especially in lignification.

## 2. Results

### 2.1. Characterization of PgrABCG Genes and the Encoded Proteins

The *PgrABCG* genes were named according to their phylogenetic relationships with *ABCG* genes in *Arabidopsis*. The PgrABCG protein varied in length from 592 to 1492 amino acids, their molecular weight varied from 64.64 to 169.51 kDa, and the isoelectric points of these proteins ranged from 6.27 to 9.84 (Table S1). The number of exons ranged from 1 to 25 (Table S1). The structure prediction results showed that PgrABCGs contained four to fifteen transmembrane helices (TMHs) (Table S1). Subcellular localization prediction results showed that most PgrABCGs were localized to the PM (Table S1). However, PgrABCG7 was localized to the PM, chloroplasts, and cytoplasm; PgrABCG39 was localized to the PM and cytoplasm; and PgrABCG24 was localized to the PM, mitochondria and nucleus, implying that these proteins may have diverse functions.

### 2.2. Phylogeny and Structure Analysis of PgrABCG Genes

**Figure 1:**
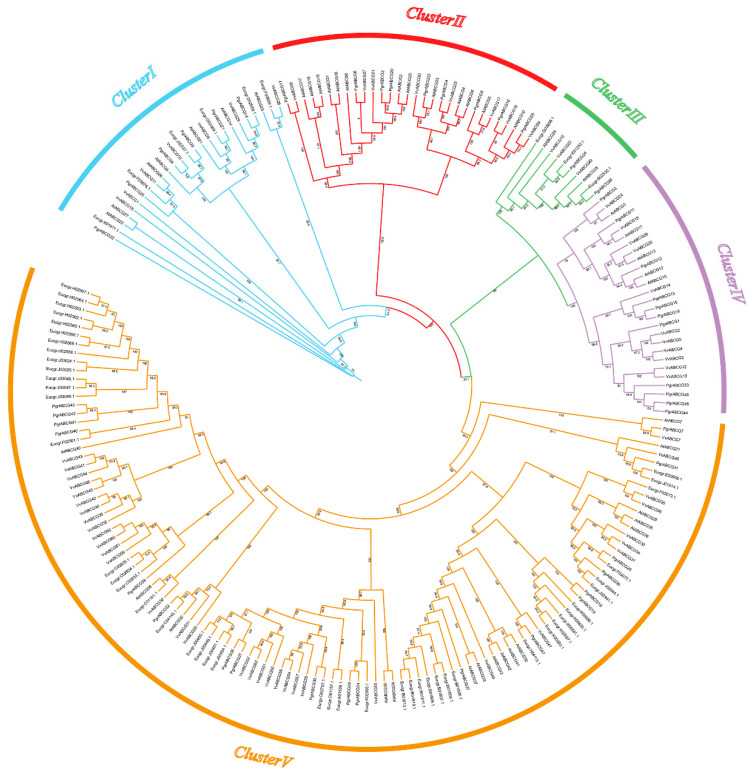
Phylogenetic analysis of ABCG proteins from pomegranate, grape, *Arabidopsis*, and eucalyptus. The tree was generated with MEGA X using the neighbor-joining method based on 1000 bootstrap replicates.

In order to reveal the phylogenetic relationship between *ABCG* genes, a total of 208 ABCG proteins from four species, including 47 from pomegranate (*Punica granatum*), 43 from *Arabidopsis thaliana*, 55 from eucalyptus (*Eucalyptus grandis*), and 63 from grape (*Vitis vinifera*) were used to construct a phylogenetic tree. All *ABCG* genes were divided into five clusters (Figure 1). Cluster III contained the lowest number of *ABCG* genes (10 genes) and Cluster V contained the greatest number of *ABCG* genes (115 genes). ABCG genes can be divided into full- and half-size molecular proteins: half-size ABCG genes comprise only one nucleotide-binding domain (NBD) and one transmembrane domain (TMD), while full-size ABCG contain two NBDs and two TMDs [25]. According to the Hidden Markov Model profiles, PgrABCG proteins consist of 17 full-size proteins and 30 half-size proteins. Interestingly, all the full-size PgrABCG proteins were distributed in cluster V, while the half-size proteins were randomly distributed in the other four clusters. Most of the *ABCG* genes between different species in the same branches had one or more homologous genes, while some had no homologous genes in other species.

The distribution of conserved motifs in PgrABCG proteins showed that most PgrABCG proteins contained motifs 1, 4, 7, and 15, and the PgrABCG proteins clustered on the same branch generally contained similar numbers and types of motifs (Figure S1A). Full-size ABCG proteins contained more motifs, and the unique motifs 8, 9, 10, 11, 12, 13, and 14 were generally distributed among the full-size ABCG proteins. The sequence information for each motif is provided in Table S2. The exon–intron structure of *PgrABCG* genes showed that the full size *PgrABCG* genes in cluster V contained more exons compared to the half-size *PgrABCG* genes in the other four clusters (Figure S1B).

### 2.3. Expression Patterns of PgrABCG Genes in Pomegranate Tissues 

**Figure 2:**
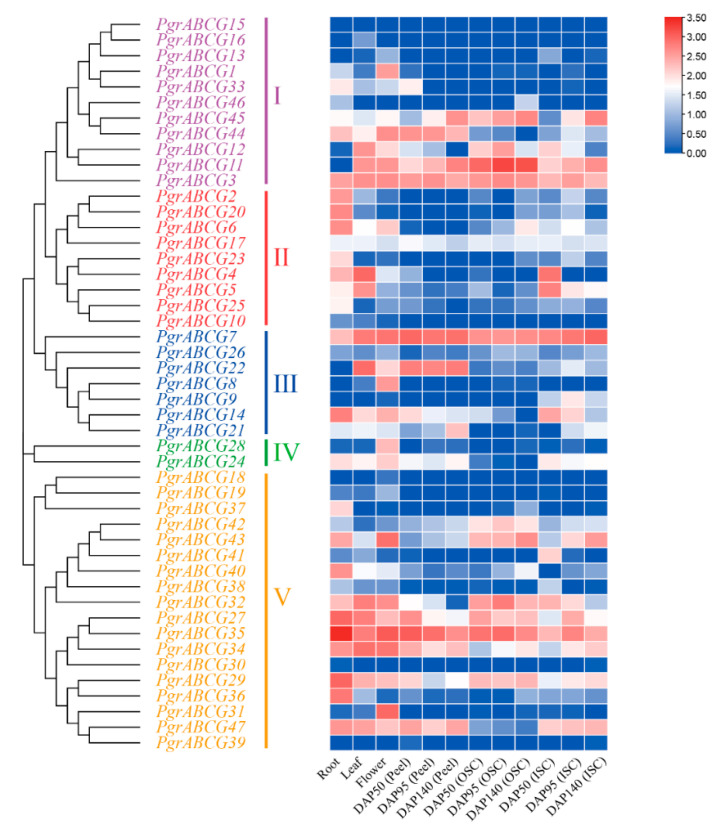
Expression profile of *PgrABCG* genes in different tissues of pomegranate based on RNA-Seq data. The transcript data were calculated with log2 normalization based on FPKM values. “DAP” = days after pollination, “ISC” = Inner Seed Coat, “OSC” = Outer Seed Coat.

**Figure 3:**
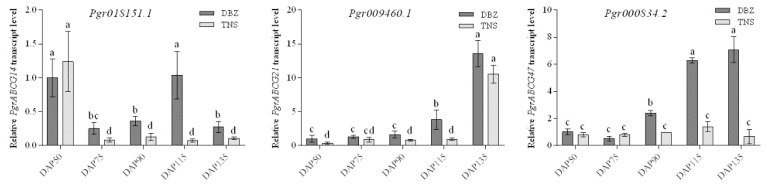
Relative expression levels of the candidate *PgrABCG* genes in the inner seed coats of hard-seeded pomegranate ‘Dabenzi’ and soft-seeded pomegranate ‘Tunisia’ at five developmental stages. “DBZ” = ‘Dabenzi;’ “TNS” = ‘Tunisia;’ “DAP” = days after pollination. Error bars indicate standard deviation (n = 3). Different letters indicate a significant difference at *p* < 0.05 using Tukey’s test.

In order to obtain more information about *PgrABCG* genes, their transcription patterns in pomegranate tissues were determined by analyzing RNA-seq data. As shown in Figure 2, several *PgrABCG* genes were specifically expressed in roots (*PgrABCG6*, *PgrABCG40*, *PgrABCG36*, *PgrABCG2*, and *PgrABCG20*), flowers (*PgrABCG31*, *PgrABCG1*, *PgrABCG28*, and *PgrABCG8*), and peel (*PgrABCG22*). Interestingly, *PgrABCG5*, *PgrABCG9*, *PgrABCG14*, *PgrABCG21*, *PgrABCG24*, and *PgrABCG47* were highly expressed in the inner seed coat, while they had very low expression levels in the outer seed coat. This suggests that these genes may function in the inner seed coat. Previous transcriptomic data showed that the relative expression levels of *PgrABCG14*, *PgrABCG21*, and *PgrABCG47* in the inner seed coat of hard-seeded pomegranate ‘Dabenzi’ were higher than those in soft-seeded pomegranate ‘Tunisia’ [40]. In this study, a qRT-PCR analysis further proved the different expression levels of these three genes in ‘Dabenzi’ and ‘Tunisia’ (Figure 3). Accordingly, these three genes were selected as candidate genes involved in seed hardness development to be further studied.

### 2.4. Co-Expression, and Evolution Analysis of the Candidate Genes

**Figure 4:**
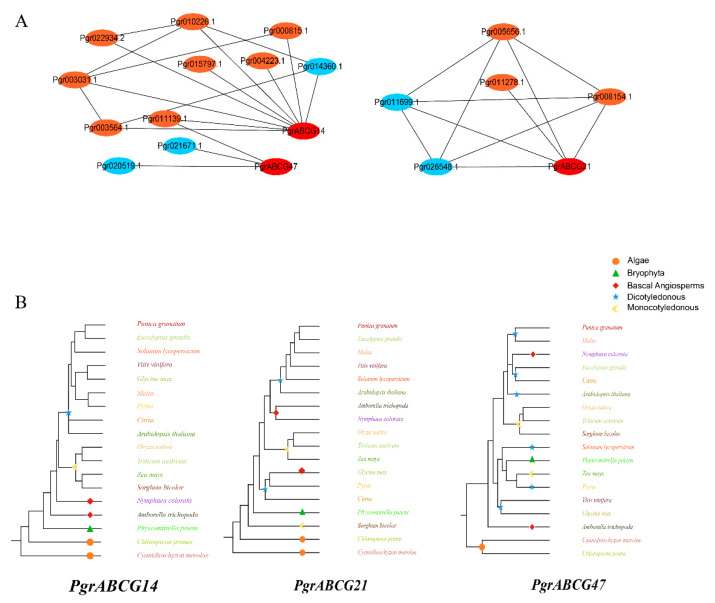
Co-expression network and evolution of the candidate *PgrABCG* genes. (**A**) The red dots represent *PgrABCG* genes, the blue dots represent genes involved in phenylpropanoid biosynthesis pathways, and the orange dots represent genes involved in hormone signaling pathways. (**B**) Rooted evolutionary trees of the candidate *PgrABCG* genes and the homologous genes in typical plant species. Different colors represent different plant species.

In order to further investigate the potential functions of *ABCG* genes in seed hardness development, we conducted WGCNA of protein-coded genes having transcriptional expression in inner seed coats with *PgrABCG* genes. The filtered genes were divided into 20 modules; the largest module contained 3641 genes and the smallest module only contained 75 genes (Figure S2A). Further, we associated the gene expression profiles in each module with all of the samples to generate a heat map of the module–sample matrix (Figure S2B). We found that the gene expression profiles in three modules, including light yellow, green, and red modules, were highly correlative to the inner seed coat (Figure S2C). Genes in these three modules were mapped in the Kyoto Encyclopedia of Genes and Genomes (KEGG) pathway. Among them, 27 and 41 genes were enriched in phenylpropanoid biosynthesis pathway and hormone signaling pathway, respectively. These genes were used to construct a co-expression network with the three candidate *PgrABCG* genes. Results showed that 16 genes were co-expressed with these *PgrABCG* genes (Figure 4A), the functional annotation of these co-expression genes was showed in Table S3. Among them, five genes are involved in lignin biosynthesis, including *Pgr021671.1* encoding Caffeic acid 3-O-methyltransferase and *Pgr014360.1* encoding shikimate O-hydroxy cinnamoyl transferase. Notably, *PgrABCG14* was highly correlated with eight genes involved in hormone signaling pathways. Among them were *Pgr011139.1* encoding isopentenyl transferase (cytokinin synthase), *Pgr015797.1* encoding cytokinin response marker protein, *Pgr000815.1* encoding auxin-responsive protein, *Pgr003031.1* encoding ABA responsive element binding factor, and *Pg022934.2* encoding brassinosteroid insensitive protein.

In order to further understand the potential functions of the three candidate *PgrABCG* genes through a plant evolutional perspective, we constructed a rooted evolutionary tree of *ABCG* genes from typical species during plant evolution. The evolution of *PgrABCG14* occurred from a sequence that originated in algae, then bryophytes, then basal angiosperms, and finally monocotyledonous and dicotyledonous plant species. This presents a highly similar trend to the origin and evolution of lignin biosynthesis during land plant evolution, wherein plant evolution took place from aquatic to terrestrial plants and from lower to higher plants (Figure 4B).

### 2.5. Functional Analysis of PgrABCG14

**Figure 5:**
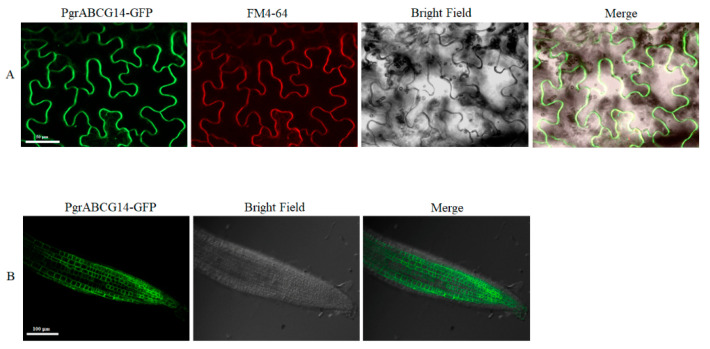
Subcellular localization of PgrABCG14-GFP fusion protein. (**A**) Subcellular localization of PgrABCG14-GFP in tobacco epidermal cells. Scale bar, 50 μm. (**B**) Subcellular localization of PgrABCG14-GFP in transgenic *Arabidopsis* plants. Scale bar, 100 μm.

**Figure 6:**
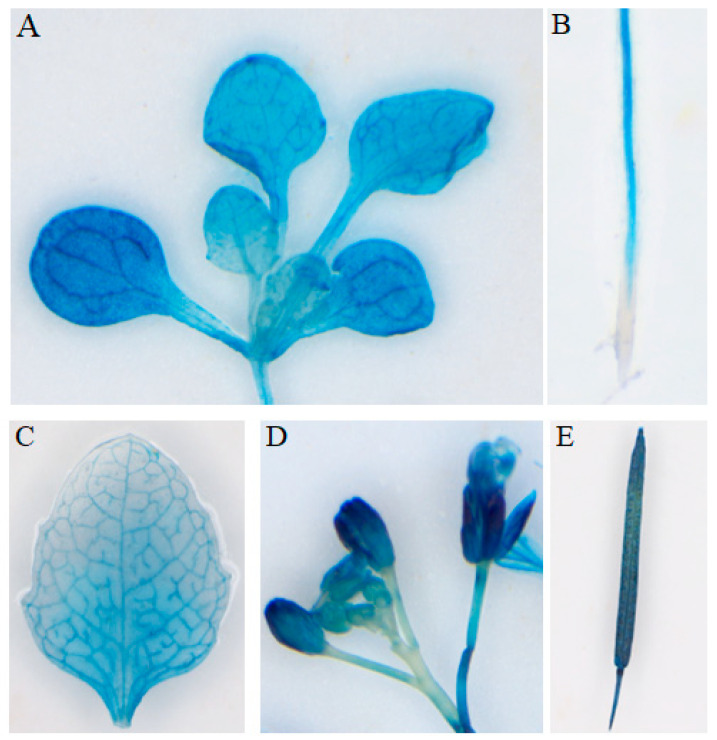
GUS activity in transgenic *Arabidopsis* plants expressing the GUS reporter gene under the control of *PgrABCG14* promoter. (**A**) 10-day rosette leaves, (**B**) root, (**C**) 45-day-old leaf, (**D**) inflorescences, (**E**) silique.

We cloned the candidate gene *PgrABCG14* and transiently expressed PgrABCG14-GFP in tobacco leaves to investigate its subcellular localization. The results showed that PgrABCG14 was localized to the PM (Figure 5A). Consistent with the results, the localization of PgrABCG14 on PM was also observed in *PgrABCG14*-*GFP* transgenic *Arabidopsis* (Figure 5B). In order to analyze the tissue-specific expression of *PgrABCG14*, we generated transgenic *Arabidopsis* plants expressing the Pro*PgrABCG14:GUS* fusion construct. GUS staining of seedlings showed that GUS activity could be observed in almost all tissue, including roots, leaves, flowers, and silique (Figure 6A–E), which was consistent with the mRNA expression results in pomegranate tissues (Figure 2). Interestingly, strong GUS activity was observed in the vascular tissues of roots (mature zones), old leaf, and petal.

**Figure 7:**
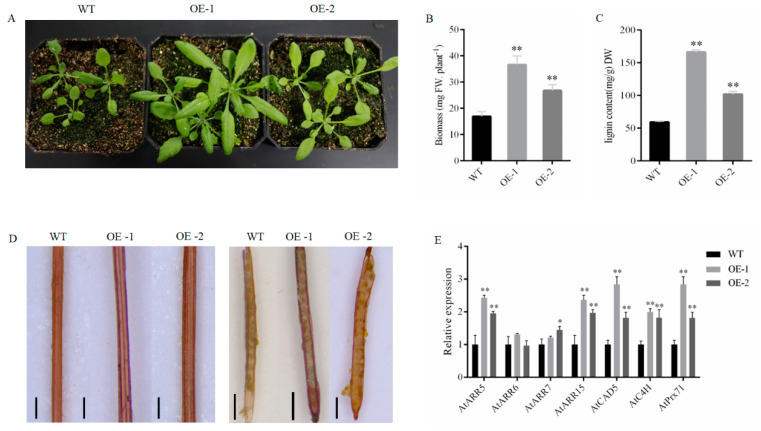
Phenotypes of *PgrABCG14* OE *Arabidopsis* plants under normal growth conditions. (**A**) Phenotypes of the wild type and *PgrABCG14* OE lines grown in soil for 4 weeks. (**B**) Biomass production of plants grown as in (**A**). (**C**) Lignin content in the shoots of plants grown as in (**A**). (**D**) Lignin staining of the stems and siliques of wild-type and *PgrABCG14* OE lines. (**E**) Expression levels of lignin biosynthesis-related genes and cytokinin response marker genes in the shoots of wild-type and *PgrABCG14* OE plants. Data are means ± SD (n = 3). Asterisks indicate values significantly different from those of the wild type (* *p* < 0.05, ** *p* < 0.01, Student’s *t* test). Bars: (**D**) 200 mm.

In order to examine the function of *PgrABCG14* in plant, we ectopically expressed *PgrABCG14-GFP* in *Arabidopsis*. Semi-quantitative PCR showed that *PgrABCG14* was successfully expressed in *Arabidopsis* (Figure S3). Two independent overexpression (OE) lines, OE-1 and OE-2, were selected for further functional analyses. Overexpression of *PgrABCG14* was able to promote *Arabidopsis* plant growth and development (Figure 7A), and shoot biomass was increased significantly in overexpressed plants (Figure 7B). Meanwhile, the lignin content was increased significantly in OE plants (Figure 7C). Notably, the deeper lignin staining in vascular tissue of stems and ventral suture of siliques were observed in OE plants compared to wild plants (Figure 7D). The transcript levels of three lignin biosynthesis related genes (*CAD5*, *C4H*, and *Prx71*) were significantly higher in the shoots of OE lines than in those of the wild type (Figure 7E).

As *PgrABCG14* is orthologous to *AtABCG14*, a gene having a clear cytokinin transport function in *Arabidopsis*, we hypothesized that *PgrABCG14* also has a role in cytokinin transport. In order to investigate the relationship between *PgrABCG14* and cytokinin, we analyzed the expression levels of four cytokinin response marker genes in the shoots of wild type and OE plants. Transcript levels of *ARR5* and *ARR15* were significantly higher in the OE plants than those in the wild type plants (Figure 7E). In addition, we exogenously applied cytokinin to the leaves of wild and OE plants. Indeed, daily spraying with 1 μM *tZ* accelerated both the WT and OE plants development, and the OE plants exhibited early bolting and stem elongation phenotypes (Figure S4).

## 3. Discussion

ABCG protein is a branch of the ABC transporter family, which was divided into ABCA-F subfamilies [41]. ABC transporters comprise four core domains: two nucleotide-binding domains (NBDs) and two transmembrane domains (TMDs). The ABC transporters are composed of two types of proteins: (i) half-size proteins, comprising only one NBD and TMD in so-called reverse orientation; and (ii) full-size proteins, containing two NBDs and two TMDs [25,42]. Half-size proteins have the capability to homodimerize and at the same time heterodimerize with another half-size proteins, thereby forming the functional ABC transporter that translocates substrates across a membrane [43]. According to the phylogenetic tree and Pfam domains of PgrABCGs, 30 full-size PgrABCGs belonged to cluster V, while 17 half-size PgrABCGs were randomly distributed to clusters I–IV, which is consistent with the phyletic evolution of ABCGs in *Arabidopsis* and grape [26,44]. Sequence analysis of *ABC* genes from different organisms suggests that half- and full-size transporters have a common ancestor and that the full-size transporters could derive from gene duplication of a half-size transporter gene [22,42,45,46]. In *Arabidopsis*, the G subfamily consists of 28 half-size transporters and 15 full-size transporters [44,45]. Some *AtABCG* genes have multiple homologs in pomegranate, while some *AtABCG* genes have no homologs in pomegranate, suggesting that gene duplication or loss may have occurred in the *ABCG* genes during evolution. We also found that the same type of PgrABCGs had similar conserved motifs, while some difference existed among conserved motifs between the two types of proteins. Full-size *PgrABCG* genes had similar numbers of exons and introns, while half-size *PgrABCG* genes contained diverse numbers of exons and introns. This is also reflected by the sequence identities among half- and full-size transporters from *Arabidopsis*, respectively.

Cell division in the seed coat accompanies the proliferation of endosperm during seed development in early stages [47]. At the early stage of pomegranate seed development, it is difficult to visually distinguish the inner and outer seed coats, and abundant cell division in the seed coat may facilitate the differentiation and expansion of the seed coat. During later stages of seed development, the outer seed coat develops into soft and juicy tissue, while the inner seed coat develops into lignified tissue. Among the *PgrABCG* genes, *PgrABCG14*, *21*, and *47* showed high expression levels in the inner seed coat, but had very low expression levels in the outer seed coat. These three *PgrABCG* genes showed higher expression levels in the inner seed coat of the hard-seeded pomegranate ‘Dabenzi’ compared to that of the soft-seeded pomegranate ‘Tunisia’. In addition, these three *PgrABCG* genes were co-expressed with the genes involved in phenylpropanoid biosynthesis pathways and hormone signaling pathways. Phytohormones, including IAA, ABA, GA, and cytokinin, have been confirmed to play important roles in regulating cell differentiation and lignin accumulation [15,16,17,18,19,20,21]. Numerous studies have shown that ABCG proteins are involved in the transport of hormones and monolignols [29,30,31,32,33,34,35]. Seed hardness formation is the result of lignin accumulation in the inner seed coat. A model was proposed that monolignols can either be transported to the cell wall to polymerize into lignin, or be glycosylated into monolignol glucosides and then stored in the vacuole [48]. Therefore, it is reasonable to presume that *PgrABCG14*, *21*, and *47* may be involved in seed hardness development through different biological processes, most likely through the lignin biosynthesis pathway and/or hormone signaling pathway.

ABCG proteins, such as AtABCG9, 14, 36, etc., are localized to the PM and are responsible for transporting specific substrates from intracellular to extracellular. Constitutive overexpression of PM-localized PgrABCG14 in *Arabidopsis* promoted plant growth and development. Its phenotype was similar to the *VviABCG14* OE plants [49]. Interestingly, we found that the lignin content in *PgrABCG14* OE plants was higher than that in WT, and the expression levels of three lignin biosynthesis-related genes (*CAD5*, *C4H*, and *Prx71*) were significantly higher in OE plants than those in the WT plants. In *Arabidopsis*, AtABCG14 is involved in the transport of *tZ*-type cytokinin from root to shoot. Mutation of *AtABCG14* results in a significant decrease in xylem and phloem cells, considerably smaller rosette leaves, shorter and thinner stems, and reduced lignin content [29,30]. In our study, we found that the expression levels of two cytokinin response marker genes (*ARR5* and *ARR15*) in *PgrABCG14* OE plants were significantly higher than that in WT plants. In addition, exogenous application of *tZ* promoted early bolting in *PgrABCG14* OE plants. The *PgrABCG14* OE plants also have longer and thicker stems compared to WT plants. It was speculated that the *PgrABCG14* gene may participate in plant development by involvement in cytokinin transport. In pomegranates, *PgrABCG14* was highly expressed in the inner seed coat, and had a higher expression level in the inner seed coats of hard-seeded pomegranate ‘Dabenzi’ than in those of soft-seeded pomegranate ‘Tunisia’. Its expression was highly correlated with that of genes which functioned in phenylpropanoid biosynthesis and hormone signaling. We proposed that *PgrABCG14* is a key candidate that is involved in seed hardness development. However, its detailed mechanisms require further study.

## 4. Materials and Methods

### 4.1. Plant Materials and Growth Conditions

Pomegranate cultivars ‘Dabenzi’ (a hard-seeded cultivar) and ‘Tunisia’ (a soft-seeded cultivar) are two of the main cultivars in China. The fruit was harvested from these two cultivars, which were grown in the Gangji Eco-agricultural Demonstration site (31°51′9.05″ N, 117°06′34.33″ E), Academy of Agricultural Sciences, Hefei, China. For gene expression, inner seed coats from ‘Dabenzi’ and ‘Tunisia’ were obtained from fruits at 50, 75, 90, 115, and 135 days after pollination (DAP) in three biological replicates. All materials were quickly frozen in liquid nitrogen and stored at −80 °C for RNA extraction.

*Arabidopsis* plants (ecotype Col-0 and transgenic *Arabidopsis* plants) were grown in soil pots in an incubator under 16 h of light (150 μmol s^−1^ m^−2^) at 22 °C, 8 h of darkness at 20 °C, and 60% relative humidity. *Nicotiana benthamiana* was grown in soil pots in an incubator under 16 h of light (500 μmol s^−1^ m^−2^) at 25 °C, 8 h of darkness at 23 °C, and 60% relative humidity.

### 4.2. Sequence Analysis of PgrABCG Genes and the Encoded Proteins

The gene and protein sequences of 47 *PgrABCG* genes were obtained according to a previous study [40]. ExPASy (https://web.expasy.org/protparam/ (accessed on 5 September 2020)) was used to calculate the basic properties of ABCG proteins. The numbers of transmembrane helices (TMHs) of the PgrABCG proteins were identified using the TMHMM Server v.2.0 (http://www.cbs.dtu.dk/services/TMHMM/ (accessed on 6 September 2020)). The subcellular localization of the PgrABCG proteins was predicted by WOLF PSORT (https://www.psort.org/ (accessed on 6 September 2020)) [50].

### 4.3. Conserved Motifs and Gene Structure Analysis

The conserved motifs of the PgrABCG proteins were identified on the MEME (http://meme.nbcr.net/meme/cgi-bin/meme.cgi (accessed on 8 October 2020)) website, with up to 15 motifs [51]. The exon–intron structures of the *PgrABCG* genes were determined by comparing of the coding sequence of each *PgrABCG* gene with its genomic sequence using TBtools v0.655 [10].

### 4.4. Phylogenetic Analysis of ABCG Proteins in Four Species

The ABCG protein sequences of grape (a species with a recent genome triplication) [52], eucalyptus (a species as the same family of pomegranate), *Arabidopsis*, and pomegranate were used to construct an unrooted tree using MEGA 7.0, based on the neighbor-joining method with bootstrap parameters of 1000 replicates [53].

### 4.5. Expression Analysis of PgrABCG Genes in Different Tissues

The abundance of *PgrABCG* transcripts in the root, flower, leaf, and the three developmental stages of the peel and seed coat (inner and outer seed coat) of ‘Dabenzi’ were collected from the RNA-seq data deposited in NCBI Sequence Read Archive (SRA) (accession SRP100581) [54]. The transcriptional abundances of *ABCG* genes were estimated using the fragments per kilobase per million (FPKM) method, and illustrated with heat map generation by TBtools v0.655 [10]. The detailed FRKM values are listed in Table S4.

### 4.6. Semi-Quantitative PCR and Quantitative Real Time Polymerase Chain Reaction (qRT-PCR) Analysis

The total RNA from pomegranate and *Arabidopsis* tissues was extracted using the RNA prep Pure Plant Kit (TianGen Bio-chemical Technology Co., Ltd., Beijing, China). First, 1 μg of RNA was used for cDNA synthesis using the Prime Script^TM^ RT Reagent Kit (TaKaRa Biomedical Technology Co., Ltd., Tokyo, Japan). The RNA samples were treated with DNase in the process of RNA extraction and cDNA synthesis. The procedures were carried out strictly following the manufacturer’s instructions. The expression levels of *PgrABCG14* in the transgenic *Arabidopsis* lines were tested by semi-quantitative PCR. The PCR program was performed as follows: 1 cycle of 95 °C for 5 min, followed by 25 cycles of 95 °C for 30 s, 59 °C for 30 s, 72 °C for 20 s, and, finally, 1 cycle of 72 °C for 5 min. qRT-PCR was carried out using SYBR Premix Ex Taq II (TaKaRa Biomedical Technology Co., Ltd., Tokyo, Japan) with an AB 7500 Fast Real-Time PCR System (Thermo Fisher Instruments Co., Ltd., Shanghai, China). The gene-specific primers were designed online using NCBI Primer-BLAST. *PgrActin7* and *AtActin2* were used as reference genes for pomegranate and *Arabidopsis*, respectively. The gene-specific primers and product sizes were listed in Table S5. The relative expression of specific genes was compared using the 2^−ΔΔCt^ method and calculated by normalizing the PCR threshold cycle (Ct) value to the expression of the reference gene. Each analysis was carried out in triplicate. Prism 6.0 was used to draw a histogram [55].

### 4.7. Co-Expression Analysis

The initial transcriptome data were collected from the RNA-seq data, which have been described above. Firstly, the genes having transcriptional expression in the inner seed coat were selected for the weighted gene co-expression network analysis (WGCNA). The parameters were as following: the weighted network was unsigned, the soft threshold power was 6, and Pearson’s correlation values (≥0.8) were used to identify the correlation between the modules and the *PgrABCG* genes. Then, the inner seed coat was used as a respective input file for the detection of significant relationships (*p* < 0.05) among the inner seed coat and the eigengene of each module. Genes were annotated to be involved in phenylpropanoid biosynthesis and hormone signaling pathways of these modules. All *PgrABCG* genes were used to construct co-expression networks using the Pearson’s correlation coefficient. Then, PCC values lower than 0.99 were removed. Networks were visualized by Cytoscape v3.7.1 [56].

### 4.8. Evolutionary Analysis of Candidate PgrABCG Genes

Algae plants including *Cyanidioschyzon merolae* and *Chloropicon primus*; bryophytes including *Physcomitrella patens*; basal angiosperms including *Amborella* and *Nymphaea*; monocotyledonous plants including *O. sativa*, *T. aestivum*, *Z. mays*, and *Sorghum bicolor*; and dicotyledonous plants including *E. grandis*, *V. vinifera*, *Solanum lycopersicum*, *Malus*, *A. thaliana*, *P. granatum*, *Glycine max*, *citrus*, and *Pyrus*, adding up to a total of 18 typical species, were selected in order to study the evolution of *ABCG* genes in the process of plant evolution. The protein sequence of each candidate *PgrABCG* gene was used for BLASTP search (with >90% coverage) to obtain the homologous protein sequences of each species using the NCBI species database (accessed on 10 November 2020). A rooted evolutionary tree of homologous ABCG proteins was constructed using DNAMAN v6.0.3.99 [57].

### 4.9. Vector Construction and Plant Transformation

The coding region of *PgrABCG14* was amplified using two primers (F, 5′-CACGGGGGACGAGCTCGGTACCATGCCTCTCCACTCCATAGCACC-3′; R, 5′-CCATGTCGACTCTAGAGGATCCCCTCAGCACCCGATGCAGTG-3′), then inserted into the pCAMBIA1300GFP vector [58]. The constructed expression vector was then used for wild type (WT) *Arabidopsis* transformation. WT transformed with the pCAMBIA1300GFP vector being used as the control plant. The T3 homozygous plants were used for phenotypic observation and functional experiment. The fragment 2.0 kb upstream of the *PgrABCG14* start codon was amplified using two primers (F, 5′- CGAGCTCGGTACCCGGGGATCCAAATGTAGAGTACTCGGAACCGATC-3′; R, 5′-ATTTACCCTCAGATCTACCATGGGGCAAAAGTAGTGGCAGTACAATT-3′) and inserted into the pCAMBIA1305 vector [59]. The constructed expression vector was transformed into WT *Arabidopsis*. The T3 homozygous plants were used for GUS histochemical analysis. All the *Arabidopsis* transformation was carried out by floral dipping [60]. The coding region of *PgrABCG14* was amplified using two primers (F, 5′-TTAAGTCCGGAGCTAGCTCTAGAATGCCTCTCCACTCCATAGCACC-3′; R, 5′-TCGCCCTTGCTCACCATGGATCCCCTCAGCACCCGATGCAGTG-3′) and inserted into the pCAMBIA1305GFP vector [59]. Then, the constructed expression vector was injected into tobacco epidermal cells by *agrobacterium tumefaciens* infection, and cultured in the dark for 3 days [61]. The expression of GFP fusion protein in cells was observed by laser confocal microscopy (Olympus Co., Ltd., Beijing, China). All of the constructed expression vectors described above were confirmed by sequencing.

### 4.10. GUS Histochemical Analysis

Histochemical staining was performed as described [62]. *Arabidopsis* plants were grown in soil pots under normal conditions. The *Arabidopsis* tissues were collected and washed twice with staining buffer [50 mM sodium phosphate buffer (pH 7.2), 0.2% Triton X-100, 2 mM potassium ferrocyanide, and 2 mM potassium ferricyanide]. Then, staining solution [50 mM sodium phosphate buffer (pH 7.2), 0.2% Triton X-100, 2 mM potassium ferrocyanide, 2 mM potassium ferricyanide, and 2 mM X-Gluc] was added to the samples. The samples were evacuated for 20 min by vacuum pump in the dark, followed by overnight incubation at 37 °C. Stained tissues were washed in increasing ethanol gradients from 50% to 100% to remove chlorophyll, and then observed with a stereomicroscope (Motic Instruments Co., Ltd., Xiamen, China).

### 4.11. Exogenous Cytokinin Treatment

The seeds were sown on Murashige and Skoog (MS) solid medium. After four leaves grew, the seedings were transferred into 1/2 Hoaglang nutrient solution (5 mM CaNO_3_, 5 mM KNO_3_, 2 mM MgSO_4_, 1 mM NH_4_H_2_PO_4_, 0.185 mM H_3_BO_3_, 2 μM [NH_4_]_6_Mo_7_O_24_, 36.6 μM MnSO_4_, 3 μM ZnSO_4_, 1.28 μM CuSO_4_, 40 μM Fe (III)-EDTA) and grown in a phytotron. After 1 week, the plants were sprayed with 1uM *trans*-zeatin (*tZ*) or 0.1% Dimethyl sulfoxide (DMSO) solution (mock) every day for the following 21 days, as Zhang et al. described [30], and then the plant phenotype was observed and photographed.

### 4.12. Lignin Histochemical Staining and Content Determination

The main stem at 1 cm above the ground and the siliques were used for staining as described [63], with the following steps: firstly, dissolve phloroglucinol in 95% absolute ethanol and 30% HCL solution (volume ratio = 1:1), the final concentration of phloroglucinol is 10%; then soak the samples to be stained in the prepared staining solution for 5 min; finally, the stained tissues were observed and photographed by stereomicroscope (Motic Instruments Co., Ltd., Xiamen, China). The deeper color in staining tissues indicated higher lignin deposition.

The determination of the plants’ lignin content was conducted as described [64], the steps as follows: firstly, 20 mg of the dry sample was added into a 15 mL centrifuge tube; then 2 mL of acetyl bromine and acetic acid was added (volume ratio = 1:1). This was kept in a constant temperature water bath at 70 °C for 30 min, and shaken every 8 min to fully mix the solution. Then, 3 mL of 2 mol/L NaOH were added, and the reaction was terminated after about 20 min. Next, 0.2 mL supernatant was placed into a new 2 mL centrifuge tube, and 3.8 mL of acetic acid was added and mixed fully. Finally, the absorbance value at 280 nm was determined.

## 5. Conclusions

*PgrABCG* genes were divided into five groups according to phylogenetic analysis. Members of Group I, II, III, and IV were half-size ABCG proteins, while group V members were full-size ABCG proteins. *PgrABCG14*, *PgrABCG21*, and *PgrABCG47* were selected as candidate genes involved in the seed hardness development according to the expression patterns, co-expression, and evolutionary analysis. Overexpression of *PgrABCG14* in *Arabidopsis* promoted plant development and lignin accumulation. This study provides a reference for understanding the characteristics and potential functions of *PgrABCG* genes.

## Data Availability

All datasets presented in this study are included in the article/Supplementary Material.

## Supplementary Materials

The following supporting information can be downloaded at: https://www.mdpi.com/article/10.3390/ijms231911661/s1.
