# Supplementary material for: Characterization of the ABC Transporter G Subfamily in Pomegranate and Function Analysis of PgrABCG14
Source: Int J Mol Sci. 2022 Oct 1;23(19):11661. doi: 10.3390/ijms231911661 (PMC9570063; doi:10.3390/ijms231911661)
Supplement: Supplementary file 1 [file ijms-23-11661-s001.zip › Table S1.pdf]

Table S1. Characteristics of *PgrABCG* genes and the encoded proteins.

Gene Name	Gene ID	Protein Length (aa)	MW (kDa)	pI	Exon Number	TMH	Subcellar location
PgrABCG1	Pgr001757.3	711	78.38	7.94	8	6	PM
PgrABCG2	Pgr001930.1	738	82.36	9.84	4	6	PM
PgrABCG3	Pgr026873.1	721	80.03	8.96	12	6	PM
PgrABCG4	Pgr023015.1	604	67.25	9.34	1	7	PM
PgrABCG5	Pgr028234.1	638	71.12	9.26	2	6	PM
PgrABCG6	Pgr025964.1	744	81.83	9.51	1	7	PM
PgrABCG7	Pgr004482.1	746	81.88	7.08	10	4	PM Chloroplast Cytoplasmic
PgrABCG8	Pgr002440.1	638	70.58	9.11	5	6	PM
PgrABCG9	Pgr008023.1	625	69.47	9.46	4	5	PM
PgrABCG10	Pgr001466.1	597	67.00	9.37	1	6	PM
PgrABCG11	Pgr005352.1	689	76.60	8.69	11	6	PM
PgrABCG12	Pgr025353.1	697	76.98	8.98	10	6	PM
PgrABCG13	Pgr008164.1	613	68.04	8.51	7	6	PM
PgrABCG14	Pgr018151.1	668	74.22	8.82	6	6	PM
PgrABCG15	Pgr008156.1	592	66.38	8.78	8	5	PM
PgrABCG16	Pgr008162.1	576	64.64	8.79	7	5	PM
PgrABCG17	Pgr013392.1	746	83.44	9.39	17	6	PM
PgrABCG18	Pgr019302.7	852	96.61	8.8	15	9	PM
PgrABCG19	Pgr021626.1	1256	142.05	8.51	21	12	PM
PgrABCG20	Pgr001931.1	754	82.91	8.56	20	7	PM
PgrABCG21	Pgr009460.1	675	75.24	9.39	7	7	PM
PgrABCG22	Pgr003186.1	760	83.68	9.09	10	5	PM
PgrABCG23	Pgr025323.4	654	73.69	8.95	2	5	PM

PgrABCG24	Pgr025550.1	1055	117.12	9.02	13	6	PM Mitochondrial Nuclear
PgrABCG25	Pgr007499.1	620	69.25	6.84	2	6	PM
PgrABCG26	Pgr007422.1	674	75.95	9.02	9	6	PM
PgrABCG27	Pgr025164.1	1394	157.64	6.56	17	13	PM
PgrABCG28	Pgr026882.1	1116	123.27	8.98	15	5	PM
PgrABCG29	Pgr012415.1	1484	167.84	8.47	22	13	PM
PgrABCG30	Pgr026142.1	1051	118.16	7.57	14	8	PM
PgrABCG31	Pgr024930.1	1475	165.19	8.73	23	11	PM
PgrABCG32	Pgr015424.1	1418	162.36	8.79	24	13	PM
PgrABCG33	Pgr008163.1	687	75.67	8.59	8	6	PM
PgrABCG34	Pgr003278.1	1401	158.71	7.16	20	13	PM
PgrABCG35	Pgr025165.1	1446	164.12	6.36	18	15	PM
PgrABCG36	Pgr005475.1	1496	169.51	7.73	22	13	PM
PgrABCG37	Pgr020172.2	1448	163.87	8.42	25	13	PM
PgrABCG38	Pgr015700.1	1451	164.99	8.81	23	13	PM
PgrABCG39	Pgr003279.1	605	69.19	6.27	8	4	PM Cytoplasmic
PgrABCG40	Pgr015047.1	1416	160.51	8.4	24	13	PM
PgrABCG41	Pgr017152.2	874	99.39	8.41	14	8	PM
PgrABCG42	Pgr001872.1	1449	163.12	8.27	24	13	PM
PgrABCG43	Pgr001873.1	1449	163.10	7.84	24	13	PM
PgrABCG44	Pgr008161.1	683	75.90	7.9	9	6	PM
PgrABCG45	Pgr008155.1	683	75.98	6.87	9	6	PM
PgrABCG46	Pgr008153.1	659	72.95	8.52	8	6	PM
PgrABCG47	Pgr000834.2	1345	152.24	8.57	22	11	PM

aa, amino acids; MW, Molecular weight; pI, isoelectric point; TMH, transmembrane helices; PM, plasma membrane.
