# Supplementary material for: Characterization of the ABC Transporter G Subfamily in Pomegranate and Function Analysis of PgrABCG14
Source: Int J Mol Sci. 2022 Oct 1;23(19):11661. doi: 10.3390/ijms231911661 (PMC9570063; doi:10.3390/ijms231911661)
Supplement: Supplementary file 1 [file ijms-23-11661-s001.zip › Table S2.pdf]

Table S2. The sequences information of each conserved motif

Motif 1	RGJSGGZRKRVSIGVEJVANPSJJFLDEPTSGLDSRAAAIVMRTLRNLAD
Motif 2	AJMGPSGAGKTTLLDVLAGRLTGG
Motif 3	SGYVEQBDILSPTLTVRETLLYSAWLRLP
Motif 4	RTVVISJHQPSPETFDLFDDJILLSEG
Motif 5	FFESMGFKCPERKGVADFLQEVTSKKDQEQYW
Motif 6	FNLFSGFFIPRPRIPKWWRWYYWISPVAWTLYGLLASZFGD
Motif 7	PVFAEERTVFYRERAAGMYSSSAYALANV
Motif 8	EMTVRETLDFSARCQGVGTRYEMLAELSRREKEAGIKPDPEIDAFMKATA
Motif 9	RLPVFYKQRDFLFYPAWAYALPTWILKIPISLLESGIWVILTYYVIGFAP
Motif 10	NKELIKELSTPPPGSKDLYFPTQYSQSFFTQCKACLWKQHWSYWRNPQYN
Motif 11	CADTMVGDEMRRGISGGQKKRVTTGEMJVGPAKALFMDEISTGLDSSTTF
Motif 12	KACFAREWLLMKRNSFVYIFKTVQJTIMALITMTVFLRTEM
Motif 13	TILQDVSGIIKPSRMTLLLGPPGSGKTTLLLALAGKLDKDL
Motif 14	TFDNVNYYVDMPAEMKEQGVEEDRLQLLRDVSGAFRPGVLT
Motif 15	SKEEKRERVEEVIEELGLDDLRBTLVGLP
