# Supplementary material for: Characterization of the ABC Transporter G Subfamily in Pomegranate and Function Analysis of PgrABCG14
Source: Int J Mol Sci. 2022 Oct 1;23(19):11661. doi: 10.3390/ijms231911661 (PMC9570063; doi:10.3390/ijms231911661)
Supplement: Supplementary file 1 [file ijms-23-11661-s001.zip › Table S3.pdf]

Table S3. The functional annotation of the genes which co-expressed with the candidate *PgrABCG* genes

Gene	KEGG Orthology
Pgr020519.1	K01850|1|3e-123|441|vvi:100266283|chorismate mutase [EC:5.4.99.5]
Pgr021671.1	K13066|1|2e-125|449|pop:POPTR_0012s00670g|caffeic acid 3-O-methyltransferase [EC:2.1.1.68]
Pgr014360.1	K13065|1|5e-40|164|rcu:RCOM_1600120|shikimate O-hydroxycinnamoyltransferase [EC:2.3.1.133]
Pgr011699.1	K14454|1|4e-160|564|egr:104436950|aspartate aminotransferase, cytoplasmic [EC:2.6.1.1]
Pgr026548.1	K00276|1|8e-141|501|sot:102600413|primary-amine oxidase [EC:1.4.3.21];K04733|2|5e-140|499|mdm:103412692|interleukin-1 receptor-associated kinase 4 [EC:2.7.11.1]
Pgr003031.1	K14432|1|1e-89|330|cic:CICLE_v10012205mg|ABA responsive element binding factor
Pgr000815.1	K14484|1|2e-49|195|fve:101293288|auxin-responsive protein IAA
Pgr003564.1	K14496|1|7e-93|339|cic:CICLE_v10022440mg|abscisic acid receptor PYR/PYL family
Pgr010226.1	K14486|1|4e-149|528|pper:PRUPE_ppa022314mg|auxin response factor
Pgr022934.2	K13415|1|2e-168|594|tcc:TCM_001284|protein brassinosteroid insensitive 1 [EC:2.7.10.1 2.7.11.1]
Pgr008154.1	K14513|1|2e-46|186|mdm:103408320|ethylene-insensitive protein 2;K09833|2|3e-33|142|cit:102609326|homogentisate phytyltransferase / homogentisate geranylgeranyltransferase [EC:2.5.1.115 2.5.1.116];K13356|3|2e-19|96.3|pda:103696395|alcohol-forming fatty acyl-CoA reductase [EC:1.2.1.84];K10395|4|8e-19|94.7|pmum:103342269|kinesin family member 4/21/27;K15086|5|1e-16|87.8|atr:18440990|(3S)-linalool synthase [EC:4.2.3.25]
Pgr008512.1	K10760|1|5e-116|417|egr:104414901|adenylate isopentenyltransferase (cytokinin synthase)
Pgr011139.1	K10760|1|4e-105|381|egr:104453788|adenylate isopentenyltransferase (cytokinin synthase)
Pgr004223.1	K10760|1|1e-98|360|vvi:100247527|adenylate isopentenyltransferase (cytokinin synthase)
Pgr011278.1	K14491|1|0.0|828|tcc:TCM_021300|two-component response regulator ARR-B family
Pgr015797.1	K14491|1|3e-82|306|csv:101215220|two-component response regulator ARR-B family
