# Supplementary material for: Characterization of the ABC Transporter G Subfamily in Pomegranate and Function Analysis of PgrABCG14
Source: Int J Mol Sci. 2022 Oct 1;23(19):11661. doi: 10.3390/ijms231911661 (PMC9570063; doi:10.3390/ijms231911661)
Supplement: Supplementary file 1 [file ijms-23-11661-s001.zip › Table S4.pdf]

Table S4. The transcriptome data (FPKM values) of *PgrABCGs* in different tissues.

gene	Root	Leaf	Flower	Peel Stage1	Peel Stage2	Peel Stage3	Outer seed coat Stage1	Outer seed coat Stage2	Outer seed coat Stage3	inner seed coat Stage1	inner seed coat Stage2	inner seed coat Stage3
Pgr001757.1 (PgrABCG1)	1.72	0.22	27.16	0.15	0	0	0	0.07	0.07	0	0.13	0
Pgr025964.1 (PgrABCG2)	40.76	4.05	10.12	0.07	0	0	0.32	0.97	5.78	2.03	3.85	1.21
Pgr026873.1 (PgrABCG3)	24.55	37.8	39.32	28.77	35.33	20.61	31.33	43.94	33.13	14.82	26.74	14.49
Pgr023015.1 (PgrABCG4)	15.63	114.32	2.35	0.86	0	0	0.16	0	0	79.66	0	0
Pgr028234.1 (PgrABCG5)	5.22	36.44	0.84	0.41	0.15	0.23	1.05	0.08	0.39	54.02	6.06	4.33
Pgr001930.1 (PgrABCG6)	29.77	0.97	0.2	0	0	0	0.32	0	0.6	0.34	1.66	0.32
Pgr004482.1 (PgrABCG7)	13.46	67.31	80.7	110.42	76.22	89.59	36.79	32.57	40.41	46.94	73.06	120.83
Pgr002440.1 (PgrABCG8)	0	0.24	29.06	0	0	0	0	0.08	0.08	0	0	0
Pgr008023.1 (PgrABCG9)	0	0	0.08	0	0	0	0	0	0	1.55	5.96	1.75
Pgr001466.1 (PgrABCG10)	0.43	0.26	0.08	0	0	0	0	0	0	0	0	0
Pgr005352.1 (PgrABCG11)	0	32.18	31.94	7.97	15.09	53.44	113.57	329.57	233.95	9.11	18.44	38.57
Pgr025353.1 (PgrABCG12)	0.07	33.26	7.75	2.15	1.1	0	9.42	26.9	2.27	9.12	2.84	0.27
Pgr008164.1 (PgrABCG13)	0	0.08	0.88	0	0	0	0	0	0	0.69	0	0.08
Pgr018151.1 (PgrABCG14)	58.33	7.75	17.51	7.68	2.88	2.28	2.01	0.51	0	22.71	8.6	1.28
Pgr008156.1 (PgrABCG15)	0	0	0	0	0	0	0	0	0	0	0	0
Pgr008162.1 (PgrABCG16)	0	0.53	0	0	0	0	0	0	0	0	0	0

Pgr013392.1 (PgrABCG17)	3.01	2.94	2.08	3.53	2.51	1.51	2.69	2.13	2.72	2.82	2.08	2.29
Pgr019302.7 (PgrABCG18)	0	0	0.17	0	0	0	0	0	0	0	0	0
Pgr021626.1 (PgrABCG19)	0.28	0.2	0.96	0	0	0	0	0	0	0.03	0	0
Pgr001931.1 (PgrABCG20)	45.68	0.34	0.06	0	0	0	0.06	0	0.59	0.56	1.12	0.06
Pgr009460.1 (PgrABCG21)	2.5	3.04	2.31	0.61	1.14	12.37	0	0	0.07	0	1.95	3.17
Pgr003186.1 (PgrABCG22)	0	101.44	8.18	59.06	49.46	63.73	0.19	0.38	0.26	1	2.6	1
Pgr025323.4 (PgrABCG23)	7.82	0.08	0.15	0	0	0	0	0	0.38	0.32	1.51	0.29
Pgr025550.1 (PgrABCG24)	7.04	4.85	9.69	3.31	2.44	4.7	0.22	0.05	0	5.66	3.36	4.07
Pgr007499.1 (PgrABCG25)	4.88	0.08	0.55	0.5	0.16	0	0.16	0.16	0.4	0.75	0.84	0.31
Pgr007422.1 (PgrABCG26)	0.61	0.38	0.8	0.08	0.29	0.22	0.43	1	0.88	0.31	0.56	0.99
Pgr025164.1 (PgrABCG27)	118.05	59.41	13.64	37.79	4.73	3.2	24.79	10.33	12.75	2.45	19.77	4.35
Pgr026882.1 (PgrABCG28)	0.09	0.09	14.12	0	0.17	0.13	0	0	0.09	0.04	0.13	0.04
Pgr012415.1 (PgrABCG29)	123.15	17.62	12.55	8.33	1.73	4.11	13.84	11.52	15.62	2.89	5.89	7.57
Pgr026142.1 (PgrABCG30)	0.05	0	0	0	0	0	0	0	0	0	0	0.04
Pgr024930.1 (PgrABCG31)	0.1	0.21	114.77	0	0	0	0.03	0	0.07	0.08	0.06	0
Pgr015424.1 (PgrABCG32)	12.8	59.67	38.23	4.11	2.18	0.07	26.46	63.15	15.63	16.64	7.65	1.36
Pgr008163.1 (PgrABCG33)	5.88	1.12	1.77	5.2	0	0	0	0	0	0	0.07	0
Pgr003278.1 (PgrABCG34)	33.93	87.87	76.07	18.63	7.15	12.7	1.25	3.39	6	1.63	5.95	9.85
Pgr025165.1 (PgrABCG35)	965.47	61.6	176.96	155.46	92.49	38.25	83.05	101.97	43.52	14.96	50.81	19.8
Pgr005475.1 (PgrABCG36)	72.09	1.04	0.1	0.41	0.1	0.13	0.03	0.03	0.82	0.64	0.65	0.41

Pgr020172.2 (PgrABCG37)	8.19	0	0.03	0	0	0	0	0.07	0.78	0	0	0.03
Pgr015700.1 (PgrABCG38)	1.21	0.45	0.46	0.04	0.03	0.03	0.07	0.03	0.03	1.58	0	0.03
Pgr003279.1 (PgrABCG39)	0	0	0	0.1	0	0	0	0	0	0	0	0.04
Pgr015047.1 (PgrABCG40)	35.95	3.67	2.61	0.58	0.17	0.34	0.2	0.87	3.1	0	0.43	0.66
Pgr017152.2 (PgrABCG41)	0.35	0.7	0.11	0.06	0	0	0	0	0	8.87	0.11	0
Pgr001872.1 (PgrABCG42)	1.36	0.14	0.47	0.85	1.24	1.77	6.39	11.05	6.31	0.92	1.95	2.12
Pgr001873.1 (PgrABCG43)	21.75	2.16	88.99	0.53	1.18	1.77	14.45	16.1	39	1.38	7.98	28.34
Pgr008161.1 (PgrABCG44)	12.23	5.13	38.31	34.98	32.18	15.56	0.52	0.32	0	0.62	2.41	1.05
Pgr008155.1 (PgrABCG45)	4.23	2.45	4.54	1.04	5.17	35.92	11.69	25.95	48.15	0.37	6.28	51.58
Pgr008153.1 (PgrABCG46)	1.16	0	0	0	0	0	0	0	1.66	0	0	0
Pgr000834.2 (PgrABCG47)	32.33	24.54	11.5	23.87	9.46	22.16	0.6	0.39	0.22	9.41	12.19	15.04
