# Supplementary material for: Characterization of the ABC Transporter G Subfamily in Pomegranate and Function Analysis of PgrABCG14
Source: Int J Mol Sci. 2022 Oct 1;23(19):11661. doi: 10.3390/ijms231911661 (PMC9570063; doi:10.3390/ijms231911661)
Supplement: Supplementary file 1 [file ijms-23-11661-s001.zip › Table S5.pdf]

Table S5. The gene-specific primers used in this study.

| gene | Sequence of forward primer | Sequence of reverse primer | Product size (bp) |
|---|---|---|---|
| *PgrABCG14* | 5'- GCCCGACCCTATCACATTTATC -3' | 5'- GTCGCCTGCTTCACATCCAT -3' | 114 |
| *PgrABCG21* | 5'- TACGAGTGTGGAAATGGGGC -3' | 5'- CAAAGCAGCAACATCCCACC -3' | 96 |
| *PgrABCG47* | 5'- TCGGACCAGTACAAGGCAAC -3' | 5'- TGTTTCCACAGACACGCAGT - 3' | 134 |
| *AtC4H* | 5'- TCGTGCCTCACATGAACCTC -3' | 5'-GCTTCCACGTGCGATTCTTC - 3' | 169 |
| *AtCAD5* | 5'- GTAATTGACACGGTGCCTGTTC -3' | 5'- GCTCCCCGTTATCACTTTCCTC - 3' | 153 |
| *AtPrx71* | 5'- CCGGCACTAGGATTGGGTTT -3' | 5'- GGATCGAACCGTCACAACCT - 3' | 162 |
| *AtARR5* | 5'- ATGGCTGAGGTTTTGCGTCC -3' | 5'- GAGACTCTGAGTAACCGCTCG - 3' | 140 |
| *AtARR6* | 5'- CAATGCCCGGTATGACTGGA -3' | 5'- TCCGAGAGTTTTACCGGCTT - 3' | 169 |
| *AtARR7* | 5'- CGGAGTTGCATGTTCTTGCC -3' | 5'- TTAGAAGCCCCTTTGCCTCC - 3' | 154 |
| *AtARR15* | 5'- AGGTGGTGAAGCTGAAGAAGG -3' | 5'- TGGAGTGTCGTCATCAAGGG - 3' | 145 |
| *PgrActin7* | 5'- TATTGTTGGTCGTCCCAGGC -3' | 5'- CCTCTCTTCGATTGGGCCTC - 3' | 90 |
| *AtActin2* | 5'- GGTAACATTGTGCTCAGTGGTGG -3' | 5'- AACGACCTTAATCTTCATGCTGC - 3' | 108 |
