# Supplementary figures and images for: Characterization of the ABC Transporter G Subfamily in Pomegranate and Function Analysis of PgrABCG14
Source: Int J Mol Sci. 2022 Oct 1;23(19):11661. doi: 10.3390/ijms231911661 (PMC9570063; doi:10.3390/ijms231911661)

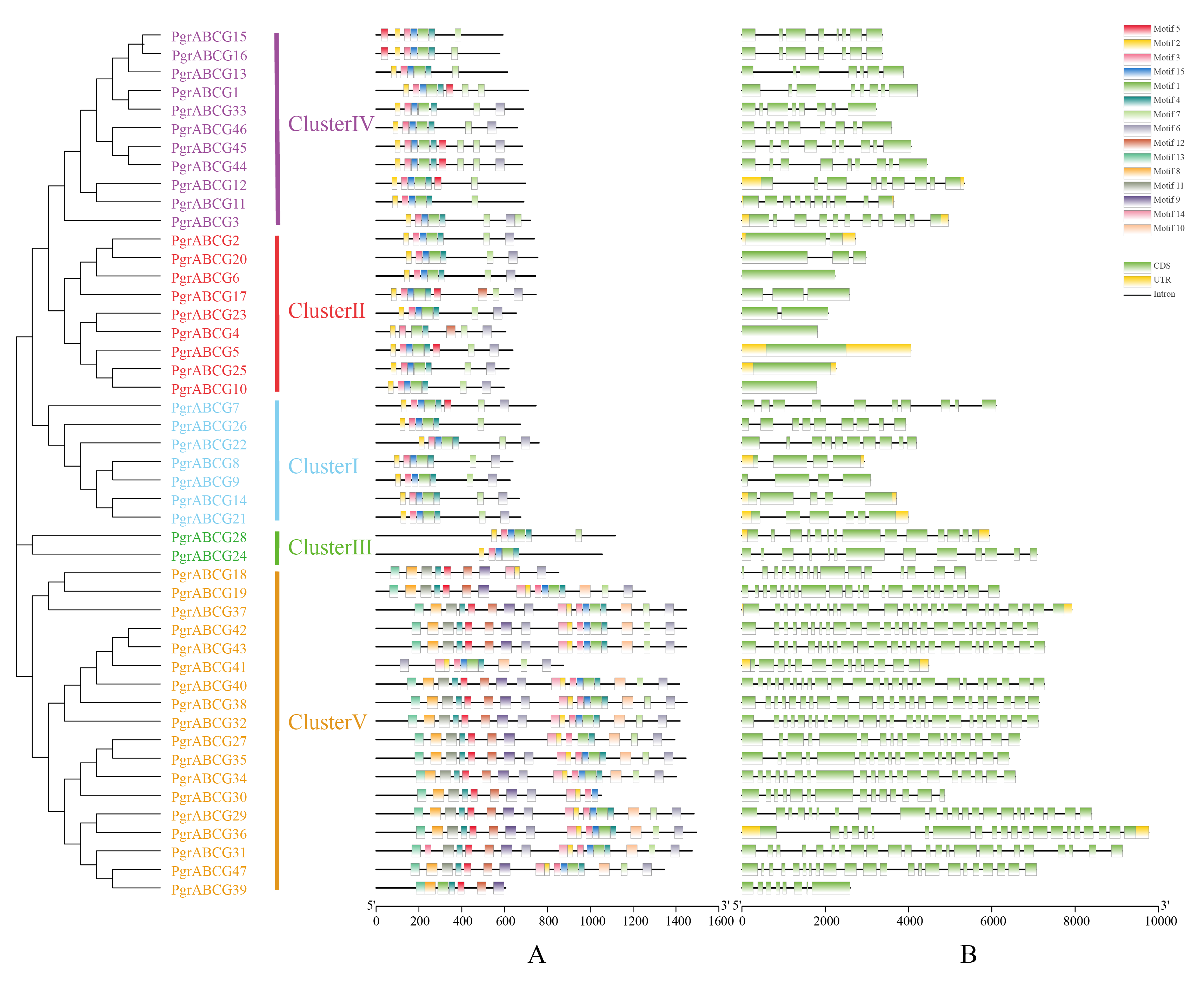

Supplement: Supplementary file 1 [file ijms-23-11661-s001.zip › Figure S1.tif]

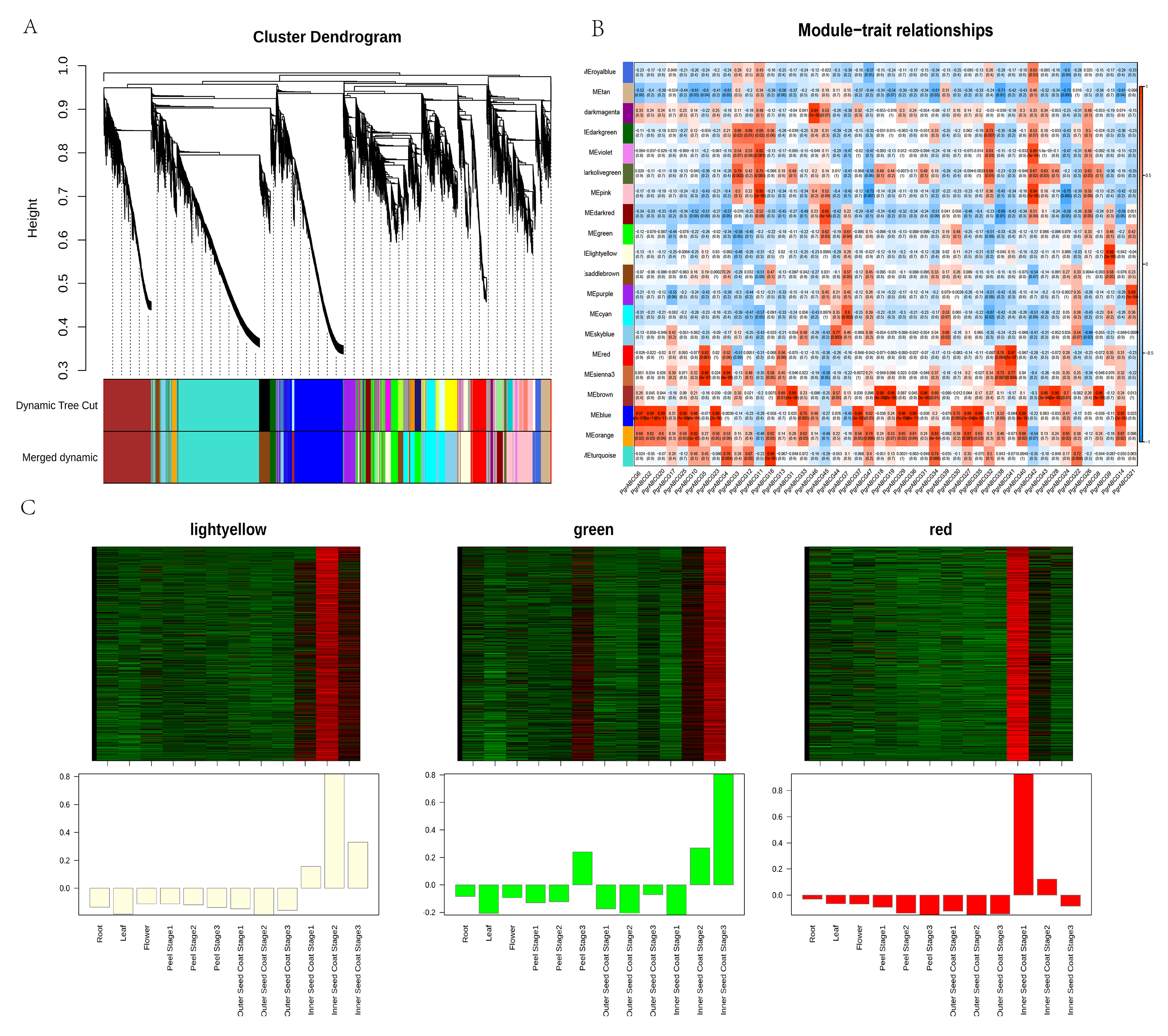

Supplement: Supplementary file 1 [file ijms-23-11661-s001.zip › Figure S2.tif]

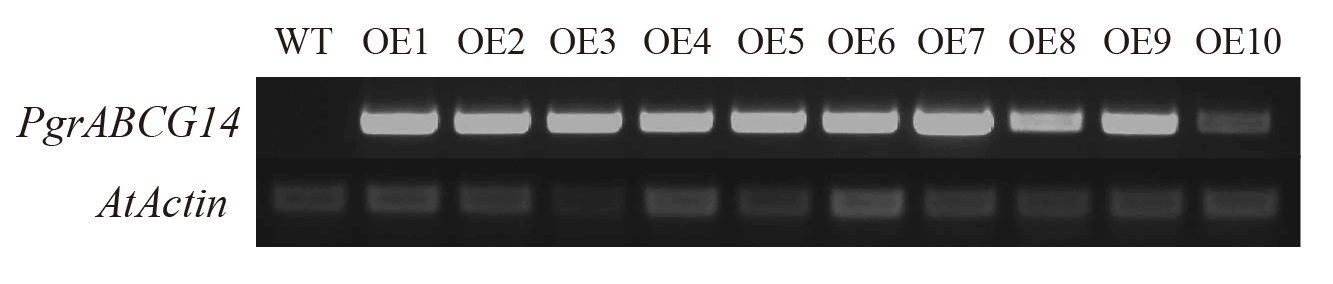

Supplement: Supplementary file 1 [file ijms-23-11661-s001.zip › Figure S3 .tif]

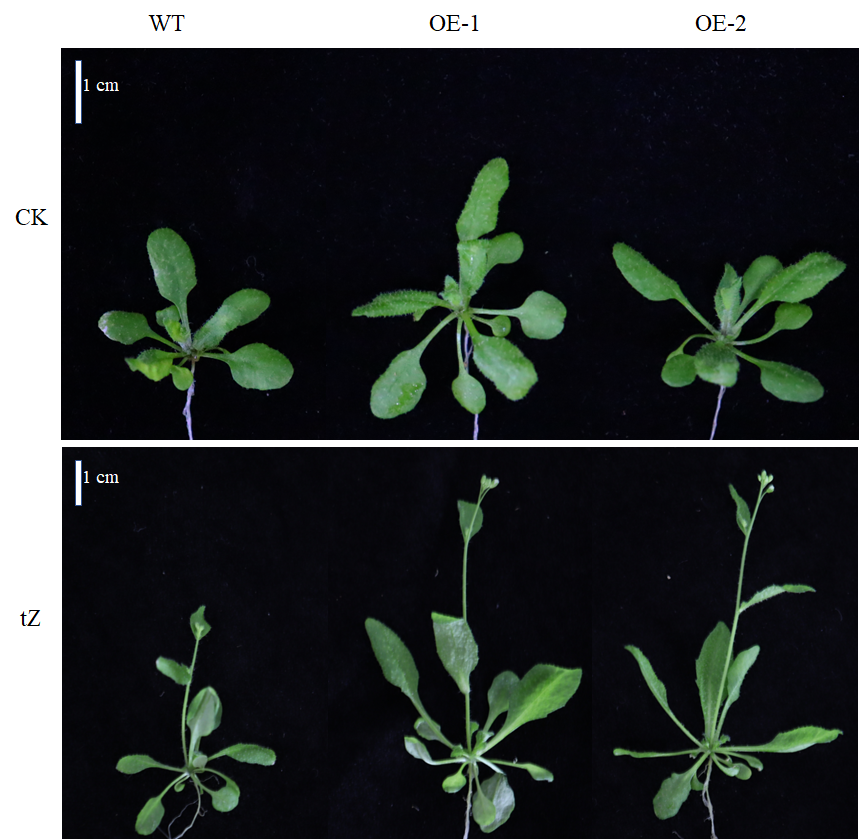

Supplement: Supplementary file 1 [file ijms-23-11661-s001.zip › Figure S4.tif]
